# Draft genome sequence of *Mycobacterium avium* complex isolated from a patient with sepsis in Thailand

**DOI:** 10.1128/mra.00063-25

**Published:** 2025-06-23

**Authors:** Panuwat Sathianpitayakul, Takeshi Komine, Panan Ratthawongjirakul, Manabu Ato, Pitak Santanirand, Hanako Fukano

**Affiliations:** 1 Department of Mycobacteriology, Leprosy Research Center, National Institute of Infectious Diseases, Higashi-Murayama, Tokyo, Japan; 2 Program of Molecular Sciences in Medical Microbiology and Immunology, Faculty of Allied Health Sciences, Chulalongkorn University 26683, Bangkok, Thailand; 3 Center of Excellence for Innovative Diagnosis of Antimicrobial Resistance, Faculty of Allied Health Sciences, Chulalongkorn University 26683, Bangkok, Thailand; 4 Microbiology Unit, Faculty of Medicine Ramathibodi Hospital, Mahidol University 26687, Bangkok, Thailand; 5 Department of Bacteriology I, National Institute of Infectious Diseases, Shinjuku, Tokyo,, Japan; University of Maryland School of Medicine, Baltimore, Maryland, USA

**Keywords:** *Mycobacterium avium*, Non-tuberculous *Mycobacterium*

## Abstract

We report the draft genome sequence of *Mycobacterium avium* complex isolate cultured from the blood of a patient in Thailand. Genomic features analysis indicates that this strain represents a distinct taxon within the MAC group.

## ANNOUNCEMENT

*Mycobacterium* spp. are acid-fast, non-motile, non-spore-forming bacilli that often appear beaded or ghost-like due to poor Gram stain uptake. Colony morphology varies by species; most are aerobic, and some are microaerophilic (1, 2). These organisms are commonly found in soil, water, and animals (3). Pathogenicity depends on bacterial characteristics as well as host and environmental factors (3).

*Mycobacterium* sp. MUNTM1 was isolated from the blood of a 45-year-old female patient with sepsis. Colonies were sub-cultured on Löwenstein–Jensen (LJ) medium at 37  °C for 10 days, and the same conditions were used for genomic DNA extraction. Antimicrobial susceptibility testing was performed by microbroth dilution according to CLSI M24 (2018) guidelines (4, 5) at the Microbiology Laboratory, Ramathibodi Hospital, Bangkok, Thailand (Table 1). Genomic DNA was extracted using the boiling method and the magLEAD® 12gC DNA extraction system (Precision System Science Co. Ltd., Japan). Library preparation was performed using the Nextera XT kit (Illumina Inc., San Diego, CA), and sequencing was conducted on the Illumina MiniSeq platform with the Mid Output kit at the National Institute of Infectious Diseases, Tokyo, Japan. This study was approved by the Ethics Committee of Chulalongkorn University (Approval No. 085/68).

**TABLE 1:** Antimicrobial susceptibility results for mycobacterial isolate^*b*^

Antimicrobial agent	MIC (μg/mL)	CLSI interpretation^*a*^
Amikacin	16	S
Clarithromycin	2	S
Linezolid	16	I
Moxifloxacin	2	I

^*a*^ S = susceptible and I = intermediate.

^*b*^ Susceptibility testing and interpretative categories were according to CLSI M24 2018 guidelines (4).

Read quality was assessed using FastQC v0.12.0 (http://www.bioinformatics.babraham.ac.uk/projects/fastqc/), which revealed no quality issues or adapter contamination. Reads were assembled *de novo* using SPAdes v4.0.0 (6) and annotated with the DDBJ Fast Annotation and Submission Tool (DFAST), which provides genome annotation and taxonomic assessment (7). Default settings were applied throughout.

A total of 636,100 paired-end reads (2 × 150 bp) were obtained. The draft genome sequence was 6,299,768 bp in length, consisting of 192 contigs, with an N50 value of 82,882 bp and an average coverage of 29×. The G + C content was 67.3%. Genome completeness was 99.86%, and contamination was 1.62%. A total of 5,864 coding sequences (CDSs), 3 rRNAs, and 56 tRNAs were identified, with a coding density of 88.2%.

Average nucleotide identity (ANI) analysis using FastANI v1.3 (8) showed the closest matches to *Mycobacterium vulneris* (92.87%) and *Mycobacterium senriense* (92.53%). Both are members of the *Mycobacterium avium* complex (MAC). *M. vulneris*, originally described as M. avium sequevar Q (9, 10), was isolated from a dog bite wound and a pediatric lymph node infection. *M. senriense* was reported from the sputum of an elderly pneumonia patient (11), but no further isolates have been documented. The ANI values of MUNTM1 fall below the 95% species delineation threshold, indicating that it represents a distinct taxon within the MAC group.

## Data Availability

The scaffold sequences of *Mycobacterium* sp. strain MUNTM1 have been deposited in the DDBJ/EMBL/GenBank databases under accession number BAAGDE010000001-BAAGDE010000192 for the chromosome. The Illumina paired-end fastq files are available in the Sequence Read Archive under accession number DRR633683.

## References

[1] Iivanainen EK, Martikainen PJ, Räisänen ML, Katila ML. 1997. Mycobacteria in boreal coniferous forest soils. FEMS Microbiol Ecol 23:325–332. doi:10.1016/S0168-6496(97)00040-8

[2] Falkinham JO. 2021. Ecology of Nontuberculous Mycobacteria. Microorganisms 9:2262. doi:10.3390/microorganisms9112262 34835388 PMC8625734

[3] Percival SL, Williams DW, Chalmers RM, Gray NF. 2013. Microbiology of waterborne diseases: microbiological aspects and risks: Second Edition, p 1–695. In Microbiol waterborne diseases, 2nd ed. Elsevier/Academic Pres, Amsterdam ;Boston.

[4] Laboratory Standards Institute. 2018. Susceptibility testing of *Mycobacteria*, *Nocardia* spp., and other aerobic actinomycetes. In CLSI document M24, 3rd ed. Clinical Standard Institute, Wayne, PA.

[5] Woods GL. 2000. Susceptibility testing for mycobacteria. Clin Infect Dis 31:1209–1215. doi:10.1086/317441 11073754

[6] Prjibelski A, Antipov D, Meleshko D, Lapidus A, Korobeynikov A. 2020. Using SPAdes de novo assembler. CP in Bioinformatics 70. doi:10.1002/cpbi.102 32559359

[7] Tanizawa Y, Fujisawa T, Nakamura Y. 2018. DFAST: a flexible prokaryotic genome annotation pipeline for faster genome publication. Bioinformatics 34:1037–1039. doi:10.1093/bioinformatics/btx713 29106469 PMC5860143

[8] Jain C, Rodriguez-R LM, Phillippy AM, Konstantinidis KT, Aluru S. 2018. High throughput ANI analysis of 90K prokaryotic genomes reveals clear species boundaries. Nat Commun 9:5114. doi:10.1038/s41467-018-07641-9 30504855 PMC6269478

[9] Mijs W, de Haas P, Rossau R, Van der Laan T, Rigouts L, Portaels F, van Soolingen D. 2002. Molecular evidence to support a proposal to reserve the designation Mycobacterium avium subsp. avium for bird-type isolates and “M. avium subsp. hominissuis” for the human/porcine type of M. avium. Int J Syst Evol Microbiol 52:1505–1518. doi:10.1099/00207713-52-5-1505 12361252

[10] van Ingen J, Boeree MJ, Kosters K, Wieland A, Tortoli E, Dekhuijzen PNR, van Soolingen D. 2009. Proposal to elevate Mycobacterium avium complex ITS sequevar MAC-Q to Mycobacterium vulneris sp. nov. Int J Syst Evol Microbiol 59:2277–2282. doi:10.1099/ijs.0.008854-0 19620376

[11] Abe Y, Fukushima K, Matsumoto Y, Niitsu T, Nabeshima H, Nagahama Y, Akiba E, Haduki K, Saito H, Nitta T, Kawano A, Tanaka M, Matsuki T, Motooka D, Tsujino K, Miki K, Nakamura S, Iida T, Kida H. 2022. Mycobacterium senriense sp. nov., a slowly growing, non-scotochromogenic species, isolated from sputum of an elderly man. Int J Syst Evol Microbiol 72. doi:10.1099/ijsem.0.005378 35604945

